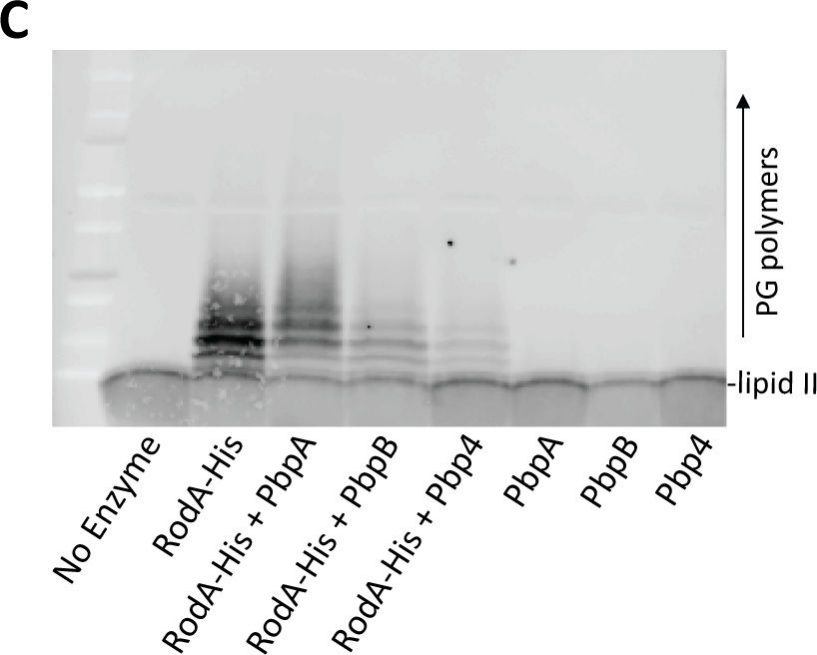# Correction for Nelson et al., “Pbp4 provides transpeptidase activity to the FtsW-PbpB peptidoglycan synthase to drive cephalosporin resistance in *Enterococcus faecalis*”

**DOI:** 10.1128/aac.00594-25

**Published:** 2025-05-20

**Authors:** Madison E. Nelson, Jaime L. Little, Christopher J. Kristich

## AUTHOR CORRECTION

Volume 68, no. 9, e00555-24, 2024, https://doi.org/10.1128/aac.00555-24. Page 6: The Elution panel in Fig. 4 should appear as shown in this correction. Regretfully we inadvertently cropped the lanes incorrectly for the anti-PbpB immunoblot when preparing the final figure file. The lanes for the anti-PbpB immunoblot are correct as shown here and do not change the conclusions as stated in the manuscript text.

**Fig 4:**
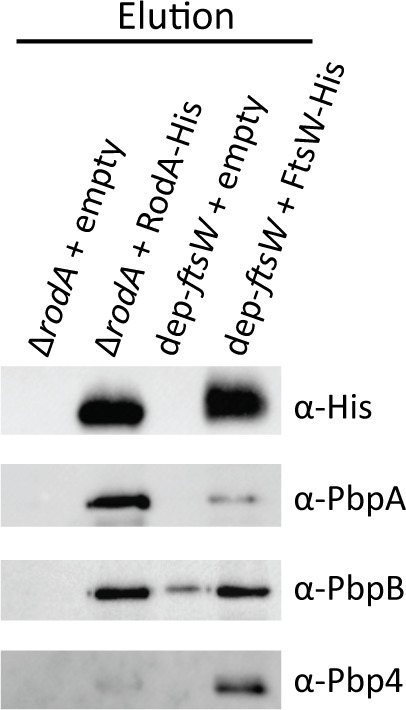


Page 7: Figure 5C should appear as shown in this correction. We inadvertently swapped the labels for two lanes in Fig. 5C when preparing the final figure file. The labels are correct as shown here and do not change the conclusions as stated in the manuscript text. We apologize for these errors, which do not impact the conclusions from the study.

**Fig 5:**